# Orthopedic Intern Curriculum for Military Medical Officers and Surgeons

**DOI:** 10.7759/cureus.116441

**Published:** 2026-09-17

**Authors:** Janse T Schermerhorn, Ben Nelson, Jacob J Avilla, Theodora C Dworak, Daniel J Song

**Affiliations:** 1 Orthopedic Surgery, Walter Reed National Military Medical Center, Bethesda, USA; 2 School of Medicine, Uniformed Services University of the Health Sciences, Bethesda, USA

**Keywords:** intern education, medical education, military, orthopedic surgery, residency

## Abstract

Introduction: Military physicians have unique challenges and responsibilities that require intentional training. The transition from medical student to orthopedic surgery intern and then to second-year resident involves a steep learning curve. The intersection of military and medical training therefore would benefit from an intentional curriculum designed to develop effective surgeons and military officers. To date, the literature describes no military-specific orthopedic intern curriculum. Materials and methods: A one-month curriculum was developed for six orthopedic interns at one military treatment facility. The curriculum was designed using Kern’s six-step approach based on an internal needs assessment. The curriculum focused on four core competency domains: taking calls, orthopedic principles, psychomotor and surgical skills, and military surgeon development. The curriculum intentionally used multiple learning modalities to ensure high fidelity, including cadaver labs, sawbone sessions, didactic lectures, interactive sessions, and more. Pre- and post-curriculum surveys were administered to the six interns to evaluate perceived confidence in content areas and to a group of faculty to assess confidence and competence in intern performance. Survey responses were scored on a five-point Likert scale and analyzed using the Wilcoxon signed-rank test (two-sided) and the Mann-Whitney U test. Open-ended questions were analyzed using thematic analysis. Results: Six interns and 23 teaching faculty (staff surgeons and senior residents) participated. Interns improved across all 16 measured clinical and technical metrics, with statistically significant improvements in six. The most pronounced gains occurred in confidence regarding independent call management (median 1.5 vs. 3.0, p = 0.031) and deployment-specific surgical procedures (e.g., amputations, fasciotomies, external-fixator placement) (median 1.5 vs. 3.0, p = 0.031). Retrospective repeat pre-curriculum survey scores were lower for several items, indicating a "response shift" and improved self-awareness of baseline deficiencies. Faculty reported high confidence in the cohort's readiness to function as competent second-year residents and strongly endorsed the military-specific curricular focus.

Conclusions: This curriculum analysis supports the value of an intentional intern curriculum that develops military officership while building orthopedic foundations. As we prepare for future conflicts, a dedicated military medical curriculum remains vital to success. Similarly, excellence as a military physician requires medical subject matter expertise. Surgeon education, therefore, should occur early and often and requires validated, comprehensive curricula to meet the needs of individual hospitals, commands, and the military as a whole. Finally, despite this curriculum’s orthopedic and military focus, we suggest the lessons learned here would be valuable to other surgical subspecialties and civilian institutions and should be replicated broadly.

## Introduction

Military physicians hold a unique dual identity as doctors and military officers. Success in each domain requires intentional effort and targeted training. As physicians begin residency training, interns confront this reality. Although true in all specialties, the learning curve is particularly steep in fields such as orthopedic surgery, where medical school provides limited instruction in many of the knowledge and skills used routinely in practice. Over the course of five clinical years and a sixth year performing research at some programs, there is a seemingly limitless amount to learn in orthopedic residency. Educational goals must therefore be established early, and priorities effectively prioritized, to achieve competency as an active-duty military officer and orthopedic surgeon.

Three orthopedic intern curricula have been published in recent years, all based on core American Board of Orthopaedic Surgery (ABOS) lessons [1-4]. Each provides a framework for an orthopedic intern curriculum, but each has strengths and weaknesses, leaving educators with options but little guidance when designing their own curriculum. To date, no military-specific curricula for orthopedics or any surgical subspecialty have been published. One military-focused curriculum in internal medicine [5] and one review [6] provide a framework from the available literature. Importantly, many military physicians feel they are inadequately prepared for the military-specific aspects of their work [7]. Here, we describe a one-month-long curriculum for orthopedic interns at a military training facility (MTF) to help meet this need.

We developed a novel, four-week immersive curriculum structured around four core competency domains: taking calls, orthopedic principles, psychomotor and surgical skills, and military surgeon development. The curriculum intentionally uses multiple learning modalities to ensure high fidelity, including cadaver labs, sawbone sessions, lectures, interactive sessions, and more. The topics span multiple essential areas of orthopedic competency, including adult reconstruction, foot and ankle surgery, hand and upper extremity surgery, sports surgery, spine surgery, and, critically, orthopedic trauma. The military surgeon’s ability to promote warfighter readiness is inextricably tied to clinical competency within their specialty. Therefore, although only certain sections of the curriculum are explicitly stated as "military focused," all provide essential training to the military orthopedic surgeon.

Although each resident at an MTF is an active-duty officer, they spend more time in scrubs than in a military uniform. Between limited exposure to battlefield casualties during peacetime and frequent rotations at civilian hospitals, military-specific skills and knowledge can deteriorate without deliberate reinforcement. Orthopedic residency is time-intensive; sustaining military-unique curricula alongside specialty training requires dedicated time and structure. This deliberate integration is increasingly important as the Military Health System (MHS) prepares physicians for contested, resource-limited environments.

To address this gap, this study aimed to describe the design and implementation of a dedicated, four-week, military-unique orthopedic intern curriculum structured around Kern's six-step framework. The secondary objective was to conduct an exploratory pilot evaluation assessing its association with changes in intern self-reported confidence, faculty-perceived competency, response-shift recalibration, and faculty feasibility.

## Materials and methods

Curriculum design and implementation

Curriculum development followed Kern's six-step approach [8]. Our needs assessment was conducted internally through a survey, informal discussions, and feedback from the previous year’s intern educational sessions. After implementation in 2015, the interns’ educational sessions underwent several subtle adjustments each year. However, they lacked several elements critical to curriculum development, such as defined goals and objectives and assessment measures. The needs assessment therefore served as a framework for structured curriculum development. Interns had substantially reduced clinical responsibilities during the one-month blocked curriculum. Each day during the four-week orthopedic intern curriculum was broken into two half-day sessions. Each session lasted about four hours but could be split into two two-hour sessions if a topic did not require a full four-hour block. Interns were given preparatory material for each session they planned to lead or teach.

The curriculum took place at several locations on the Walter Reed National Military Medical Center campus, as well as off-site locations. We utilized classrooms for sawbone labs and lectures. The medical school associated with our hospital provided a cadaver lab for several of our surgical sessions. Other cadaver labs were performed at third-party off-site locations.

Participants and curriculum survey analysis

A single-institution, mixed-methods pre- and post-survey evaluation of a dedicated "Intern Modules" educational month in an orthopedic surgery residency program was performed. Participants included all orthopedic surgery interns completing Intern Modules (n = 6) and orthopedic staff and senior residents (postgraduate years (PGY) 4-6) who participated in intern education. Interns completed an initial pre-modules survey and a post-modules survey. To assess response-shift bias (recalibration of perceived baseline competence after exposure to training), interns also completed a repeat "pre-" survey administered two weeks into the month, asking them to retrospectively rate their perceived baseline knowledge/competence using the same constructs after obtaining partial exposure to the curriculum. Teaching faculty (consisting of staff surgeons and senior residents) completed a pre-modules survey assessing perceived intern deficiencies, expectations, and priorities, and a post-modules survey assessing perceived intern achievement and the feasibility/burden of teaching for that month. Walter Reed National Military Medical Center approved the study under WRNMMC-EDO-2026-1476.

Primary outcomes were changes in interns’ self-assessed knowledge and confidence across 16 aligned competency items rated on five-point Likert scales (1 = not at all; 5 = very thoroughly/very confident). Items included subspecialty procedural understanding (adult reconstruction, arthroscopy, trauma, bedside hand procedures, elbow procedures, foot/ankle, and pediatric orthopedics) and broad military and orthopedic topics (open surgical skills, arthroscopic skills, anatomy/approaches, manual dexterity/instrument handling, call consult management confidence, confidence functioning as a competent PGY-2, surgical knowledge in a deployed setting, research methodology knowledge, and military administrative knowledge).

Secondary outcomes included intern and faculty attitudes toward a blocked-off educational month versus a longitudinal curriculum, faculty-reported feasibility/impact on clinical schedules and perceived value, and qualitative themes from open-ended responses on curriculum strengths and gaps.

Data processing and analysis

Survey response exports were reviewed for completeness. Likert-scale items were identified as numeric responses constrained to one to five and were treated as ordinal data. Free-text responses were retained verbatim and categorized into thematic bins. Intern pre- and post-curriculum responses were analyzed as paired data (n = 6) using the Wilcoxon signed-rank test (two-sided). For each item, pre- and post-survey distributions were summarized using medians (interquartile ranges (IQR)) and means (standard deviations). Median paired change (post-survey minus pre-survey value) was also summarized along with the proportion of interns demonstrating improvement or worsening of their scores. Response-shift bias was evaluated by comparing original pre-responses with repeat pre-responses (paired) using Wilcoxon signed-rank tests, reporting the magnitude and direction of shifts (repeat pre-responses minus original pre-survey value).

Because faculty surveys were anonymous and unlinked across time points (23 respondents pre-curriculum vs. 17 post-curriculum), differences between pre- and post-curriculum faculty assessments were evaluated using the independent-samples Mann-Whitney U test. To minimize survey fatigue among supervisory surgeons and senior residents, the faculty instrument evaluated eight consolidated core competency domains rather than the 16 granular procedural items completed by interns. Categorical items were summarized using counts and percentages.

Open-ended responses were consolidated using a structured thematic binning approach based on curriculum goals and common competency domains. Themes included call consult management, operating room (OR) setup, casting/splinting, anatomy/approaches, procedural skills (open and arthroscopic), trauma/deployed surgery content, research/administrative content, and requests for additional hands-on/cadaver time. Responses could be assigned to more than one theme. Analyses were conducted using Python version 3.14.3 (Python Software Foundation, Beaverton, OR, USA) utilizing the SciPy library (scipy.stats module). Statistical tests were two-sided with alpha set at 0.05. Given the small cohort (n = 6) and ordinal Likert data, we reported paired median changes and IQR alongside exact p-values to illustrate the direction and magnitude of shifts across all 16 domains. These descriptive distributions emphasize that, at n = 6, these metrics serve as exploratory measures of magnitude rather than definitive population effect sizes.

## Results

Logistics of curriculum implementation and participant demographics

This MTF orthopedic intern class consisted of six individuals: four males (n = 4, 66.7%) and two females (n = 2, 33.3%). Interns completed 100.0% of the pre- and post-curriculum assessments (n = 6). The faculty survey was sent to 22 staff surgeons and 18 senior residents. Of the 40 faculty members, 23 completed the pre-curriculum survey (57.5%), and 17 completed the post-curriculum survey (42.5%). The curriculum took place over one month, with sessions divided into morning and afternoon sessions (Table 1).

**Table 1:** Military orthopedic intern curriculum, 2026 AM: morning (generally 0800-1200), PM: afternoon (generally 1230-1630), SCH: supracondylar humerus, ORIF: open reduction internal fixation, Ex-Fix: external fixation, TKA: total knee arthroplasty, THA: total hip arthroplasty, IMN: intramedullary nail, ED: emergency department, OI: osseointegration, AAR: after action report

Date	Day	Session	Curriculum activity	Domain
Jan 12	Monday	AM	Intro + prep lecture	Military surgeon development, orthopedic principles
		PM	OR setup	Orthopedic principles
Jan 13	Tuesday	AM	Suture lab	Orthopedic principles
		PM	Splinting/casting	Taking call
Jan 14	Wednesday	AM	Global surgery, ORIF principles	Military surgeon development, orthopedic principles
		PM	Ex-Fix and fasciotomies	Military surgeon development
Jan 15	Thursday	AM	Foot and ankle lab	Psychomotor/surgical skill
		PM	Foot and ankle lab	Psychomotor/surgical skill
Jan 16	Friday	AM	Orthopedic oncology basics	Orthopedic principles
		PM	Residency academics, OI + prosthetics	Military surgeon development, orthopedic principles
Jan 19	Monday	AM	Holiday	Holiday/other
		PM	Holiday	Holiday/other
Jan 20	Tuesday	AM	Extremity war injury symposium	Military surgeon development
		PM	Spine lab	Psychomotor/surgical skill
Jan 21	Wednesday	AM	Splinting/casting	Taking call
		PM	Lower extremity amputations	Military surgeon development
Jan 22	Thursday	AM	Leadership principles	Military surgeon development, orthopedic principles
		PM	Teaching and learning principles	Military surgeon development, orthopedic principles
Jan 23	Friday	AM	Research principles	Orthopedic principles
		PM	Residency academics, MRI reading, military finance	Military surgeon development, orthopedic principles
Jan 26	Monday	AM	Call principles and cases	Taking call
		PM	Splinting/casting	Taking call
Jan 27	Tuesday	AM	Arthroscopy lab	Psychomotor/surgical skill
		PM	Arthroscopy lab	Psychomotor/surgical skill
Jan 28	Wednesday	AM	Call principles and cases	Taking call
		PM	TKA lab	Psychomotor/surgical skill
Jan 29	Thursday	AM	Upper extremity amps + fasciotomies	Military surgeon development
		PM	ED hand procedures	Taking call
Jan 30	Friday	AM	THA lab	Psychomotor/surgical skill
		PM	Team building	Holiday/other
Feb 2	Monday	AM	Call principles and cases	Taking call
		PM	“Flex” day	Holiday/other
Feb 3	Tuesday	AM	Pediatrics call principles	Taking call
		PM	Flexinail and SCH pinning	Psychomotor/surgical skill
Feb 4	Wednesday	AM	IMN lab	Psychomotor/surgical skill
		PM	IMN lab	Psychomotor/surgical skill
Feb 5	Thursday	AM	Sawbones competition	Orthopedic principles
		PM	Cadaver lab leftover	Psychomotor/surgical skill
Feb 6	Friday	AM	Curriculum AAR	Holiday/other
		PM	Residency academics, hospital research support	Orthopedic principles

A total of 56 hours were dedicated to cadaveric training, 10 hours to sawbones, 40 hours to didactic lectures, 12 hours to research discussions, and 28 hours to other hands-on tasks (suturing, splinting, etc.). Nine of the 17 faculty (n = 9, 52.9%) who responded to the post-curriculum survey had protected or available time in their schedules for teaching. They did not have to alter their clinical schedules. Six faculty (n = 6, 35.3%) had to cancel or reschedule clinical duties for their teaching responsibilities. Nine (n = 9, 39.1%) and six (n = 6, 26.1%) of the 23 faculty who responded to the pre-curriculum survey preferred or were neutral to the blocked-off month curriculum, respectively, as opposed to a year-long longitudinal curriculum. Five interns (n = 5, 83.3%) preferred the blocked-off month, and one intern (n = 1, 16.7%) was neutral.

Pre-curriculum needs assessment and faculty survey

The curriculum needs assessment was based on interns’ and faculty pre-curriculum surveys. When faculty were given a free-text option for important objectives they wanted the interns to cover over the one-month period, 14 of 22 faculty specified call consult (n = 14, 63.6%), 12 specified anatomy and surgical approaches (n = 12, 54.5%), eight specified splint/cast immobilization (n = 8, 36.4%), and seven referenced content specific to their subspecialty (n = 7, 31.8%). When asked which skill they would prioritize for mastery for the interns, the most common responses were manual skills (suturing, reductions, etc.; n = 9, 39.1%), followed by logistical efficiency (consult management, OR setup, etc.; n = 7, 30.4%), and medical knowledge (anatomy, pathology, etc.; n = 5, 21.7%). Finally, the skill most commonly lacking in recent years’ interns was understanding of anatomy and surgical exposures (n = 7, 30.4%), followed by OR setup and equipment knowledge (n = 5, 21.7%).

Interns’ self-reported confidence and perceived proficiency

Interns reported their pre- and post-curriculum confidence and perceived proficiency across 16 topics (Table 2).

**Table 2:** Interns’ pre- and post-curriculum perceived confidence in various topics * Statistically significant p-value, + IQR Ex-fix: external fixation, PGY: postgraduate year, IQR: interquartile range

Domain/survey metric	Pre-curriculum median (IQR)^+^	Post-curriculum median (IQR)^+^	p-value
Taking call			
Confidence in independent call management	1.5 (1.0)	3.0 (0.8)	0.031*
Emergency department hand procedures (steps and goals)	1.5 (1.0)	3.5 (1.0)	0.034*
Orthopedic principles			
Research methodology and administrative requirements	2.0 (0.8)	3.0 (0.8)	0.063
Open surgical procedure proficiency	2.0 (2.0)	3.5 (1.8)	0.059
Arthroscopic surgical proficiency	1.0 (0.8)	3.0 (0.8)	0.031*
Psychomotor and surgical skills			
Surgical proprioception, dexterity, and handling	2.5 (1.0)	3.0 (0.0)	0.102
Surgical anatomy and approaches	2.0 (0.8)	3.0 (1.5)	0.059
Adult reconstruction (steps and goals)	2.0 (0.8)	3.0 (1.5)	0.038*
Elbow procedures (steps and goals)	1.5 (1.0)	2.0 (0.8)	0.046*
Orthopedic trauma (steps and goals)	2.5 (1.0)	3.0 (0.8)	0.059
Foot and ankle procedures (steps and goals)	1.0 (1.5)	2.5 (1.8)	0.059
Pediatric orthopedic procedures (steps and goals)	2.0 (0.0)	3.0 (0.8)	0.102
Military surgeon development			
Deployed military surgeries (amputations, Ex-fix)	1.5 (1.0)	3.0 (1.5)	0.031*
Military administrative aspects	1.5 (2.5)	3.0 (0.8)	0.157
Overall readiness assessment			
Confidence to function as a competent PGY-2	2.0 (0.8)	3.0 (0.8)	0.063

At least half of the interns (n = 3, 50.0%) reported improved confidence and proficiency across all topics. For adult reconstruction steps and goals, arthroscopic steps and goals, and call consult management, 100.0% of interns (n = 6) improved from pre- to post-curriculum. Perceived confidence and proficiency decreased for only one intern regarding a single topic: military administrative requirements (n = 1, 16.7%). Statistically significant improvements in pre- and post-curriculum confidence and proficiency were observed in emergency department hand procedures (median 1.5 vs. 3.5, p = 0.034*), deployed military surgeries (median 1.5 vs. 3.0, p = 0.031*), arthroscopic surgical proficiency (median 1.0 vs. 3.0, p = 0.031*), adult reconstruction steps and goals (median 2.0 vs. 3.0, p = 0.038*), elbow procedure steps and goals (median 1.5 vs. 2.0, p = 0.046*), and independent call management (median 1.5 vs. 3.0, p = 0.031*).

Response-shift analysis

None of the repeat pre-curriculum responses were significantly different from the original pre-curriculum survey responses. However, intern responses decreased for seven of 16 questions (43.8%), suggesting interns may have initially overestimated their baseline knowledge and confidence in several content areas before beginning the curriculum. Only one pre-curriculum response increased in the repeated survey compared with the original, and the difference was not statistically significant (confidence in ability to manage call patients; median 1.5 vs. 2.0; p = 0.160).

Faculty confidence and assessment of intern proficiency

Faculty ratings of interns’ proficiency improved in most topics from pre- to post-curriculum. Importantly, topics considered critical in the faculty pre-curriculum needs assessment, such as knowledge of anatomy and surgical approaches (median 2.0 vs. 3.0; p = 0.002*) and independent call management (median 2.0 vs. 3.0; p < 0.001*), demonstrated statistically significant improvements. The only measured category that did not reach statistical significance was overall PGY-2 readiness (median 3.0 vs. 4.0; p = 0.382). Despite this, 10 of 17 responding faculty (n = 10, 58.8%) were confident in the interns’ ability to function as PGY-2 residents, as determined by the number of 4 (agree) and 5 (strongly agree) Likert-scale responses. This was the highest absolute percentage of 4 and 5 responses among the survey questions in Table 3.

**Table 3:** Faculty pre- and post-curriculum assessment of intern competency in various topics * Statistically significant p-value, + military responsibilities including administrative requirements, military finance, roles as an officer, and military-surgeon relevant surgeries, ++ IQR PGY: postgraduate year, IQR: interquartile range

Domain	Assessment metric	Pre-curriculum median (IQR)^++^	Post-curriculum median (IQR)^++^	p-value
Taking call	Independent call management	2.0 (0.5)	3.0 (0.0)	<0.001*
Orthopedic principles	Research methodology and administration	2.0 (1.0)	3.0 (1.0)	<0.001*
	Basic open procedure proficiency	2.0 (2.0)	3.0 (1.0)	0.001*
	Basic arthroscopic proficiency	2.0 (1.5)	3.0 (0.0)	0.002*
Psychomotor and surgical skills	Surgical proprioception and dexterity	2.0 (1.0)	3.0 (0.0)	<0.001*
	Anatomy and surgical approaches	2.0 (2.0)	3.0 (0.0)	0.002*
Military surgeon development	Overall military responsibilities^+^	2.0 (1.0)	3.0 (1.0)	<0.001^*^
Overall assessment	PGY-2 readiness confidence	3.0 (1.0)	4.0 (1.0)	0.382

Thematic analysis

When interns were asked which topic they felt least ready for in their PGY-2 year, three of six (n = 3, 50.0%) identified consult management pre-curriculum, whereas only one of six (n = 1, 16.7%) reported this post-curriculum. Among interns and faculty, the most commonly requested topic for greater coverage was call consult management (interns: 2/6, 33.3%; faculty: 3/13, 23.1%). Several individuals in both cohorts reported no content gaps that needed addressing (interns: 2/6, 33.3%; faculty: 6/13, 46.2%).

## Discussion

The curriculum described in this study was associated with improvements in interns’ self-perceived confidence and faculty-reported assessments across several essential content areas. This curriculum fulfills several unmet needs within orthopedic intern and military-specific surgeon education. First, no military-specific curriculum for orthopedic residents or interns has been published to date. Second, few orthopedic intern-specific curricula have been published previously. Each curriculum has strengths and weaknesses, but medical education is not "one-size-fits-all." Orthopedic residency programs differ in mission, goals, geographic location, and stakeholder values and therefore require curricula designed to meet their needs. In our unique MHS environment, this concept is particularly pertinent. Finally, by assessing both intern and faculty perceptions of implementation and feasibility, this curriculum provides an initial framework that may warrant replication and tailored adaptation at other training institutions.

Three notable civilian intern orthopedic curricula have been published previously [1-4]. Each of these provides a framework and point of reference for building a curriculum for this level of orthopedic trainee. The ABOS provides a surgical skills module that Westerlind et al. [4] operationalized and initially described in the curriculum in 2014, and Ford et al. [2] slightly modified in 2016. Although comprehensive, these models focus primarily on simulation and surgical technique, which are widely utilized in orthopedic training [9], but notably leave out many other essential non-technical proficiencies required of the orthopedic intern, such as clinical reasoning, individualized health system navigation, principles of managing call consults, preoperative planning, leadership, and administrative workflows. Dagher et al. [1] recently expanded on these technical foundations by introducing a month-long boot camp that evaluates competency rather than participant satisfaction alone.

Our findings align with and expand upon this existing literature. While confirming the value of simulation and laboratory-based procedural instruction documented by Westerlind et al. [4]. Ford et al. [2], our study broadens the curricular scope by integrating non-technical proficiencies, such as independent call triage, hospital administration, and multidisciplinary communication. Furthermore, our study addresses a distinct void in the surgical literature. Whereas military-specific curricula have been evaluated in non-surgical fields such as internal medicine (Roop et al. [5]) or reviewed broadly at the graduate medical education policy level (Baird et al. [6], Neuman et al. [7]), no prior surgical curriculum has incorporated military-unique operational competencies alongside civilian subspecialty standards. Our data demonstrate that active-duty trainees reported substantial, statistically significant gains in operational readiness procedures (e.g., deployed surgeries such as fasciotomies and external fixation, p = 0.031*), demonstrating that dedicated operational training can be successfully embedded into specialty residency education.

Our findings also demonstrate a meaningful improvement in intern self-efficacy regarding acute clinical responsibilities. Interns reported being substantially more prepared to triage and manage call consult patients following this curriculum. Given the steep increase in independent on-call responsibilities between the intern and junior resident years, alongside documented deficits in undergraduate musculoskeletal education in medical school [4,10], structured triage training may be educationally valuable. Teaching faculty identified call consult management as the single largest baseline knowledge gap, reinforcing that an intern curriculum lacking structured call training is incomplete. Consequently, educational time should not only be spent practicing safe reduction and immobilization techniques, as highlighted in prior models [2,4], but should also incorporate simulated, case-based call discussions, as in our curriculum.

The response-shift analysis between the interns' initial pre-curriculum survey and the repeat pre-survey provides valuable pedagogical insight. The downward shift in intern scores across seven of 16 items after two weeks of curricular exposure highlights a recognized phenomenon in medical education: novice learners frequently lack awareness of baseline deficiencies (unconscious incompetence) [11]. The subsequent post-curriculum gains across all domains suggest that the curriculum facilitated greater learner self-awareness alongside improved perceived proficiency.

The military medical officer must balance multiple responsibilities to effectively support the mission of the Defense Health Agency [12]. This includes sustaining a medically ready force, preparing for operational deployment, and maintaining high clinical competency. For orthopedic surgeons, readiness requires maintaining broad surgical capability in resource-limited, austere environments [13]. Without protected time and deliberate instruction, these military-specific competencies can atrophy during routine peacetime garrison practice and civilian rotations [14]. By dedicating 43 hours of military-specific instruction alongside core orthopedic fundamentals, our curriculum sought to actively mitigate this operational skill decay. Furthermore, because many operational surgical competencies (such as vascular shunting, fasciotomy, and external fixation) are shared across surgical specialties, this framework is readily translatable to general surgery and other surgical training programs across the MHS.

This study has several important limitations. First, the evaluation was conducted as a single-arm, uncontrolled pilot study with a small cohort of six interns at a single military treatment facility, which limits generalizability and precludes causal attribution. Without a concurrent control group or historical comparison cohort, observed gains may partially reflect normal trainee maturation, concurrent clinical exposure, or a Hawthorne effect from focused faculty attention. Second, all outcome measures relied on subjective intern self-reports and unblinded faculty impressions rather than objective, psychometrically validated performance assessments (such as Objective Structured Assessments of Technical Skills (OSATS)). Third, survey instruments were developed internally and lack formal psychometric validation or internal consistency reliability testing (e.g., Cronbach's alpha). Fourth, because supervisory faculty served as both instructors and evaluators, ratings may have been subject to social desirability or courtesy bias. Fifth, faculty survey response rates dropped from 57.5% pre-curriculum to 42.5% post-curriculum, introducing potential non-response bias. Sixth, the authors ran multiple statistical comparisons on a small sample without family-wise error-rate corrections, increasing the risk of type I error. Finally, 35.3% of faculty had to adjust or cancel clinical responsibilities to deliver instruction, highlighting a logistical and productivity burden that must be weighed against subjective educational gains in resource-constrained military facilities. Future multicenter studies incorporating objective skills testing and longitudinal follow-up are warranted to evaluate skill retention and clinical efficacy.

## Conclusions

Implementing a dedicated, four-week orthopedic intern curriculum that combined foundational surgical principles with military-specific operational instruction was associated with marked improvements in intern self-reported confidence and faculty-perceived readiness. By combining a targeted needs assessment with multi-modal simulation and clinical vignettes, this pilot model addressed transition gaps in call management, surgical exposures, and deployed trauma care that standard residency orientations often overlook.

While designed to address the specific operational requirements of active-duty military surgeons, the educational framework described provides a useful template for surgical training programs seeking to formalize intern boot camps. Early, protected educational blocks focused on practical skills, clinical triage, and institutional workflows offer a promising model that warrants further multi-institutional investigation incorporating objective competency metrics and long-term outcome assessments.

## Appendices

**Table 4:** Intern survey instruments * Intern core competency matrix response scale: 1 = not at all confident; 2 = slightly confident; 3 = moderately confident; 4 = very confident; 5 = completely confident. Administration note: The 16 core competency items in Section 2 were completed at three time points: baseline (pre-curriculum), retrospectively at two weeks to assess response-shift bias (repeat pre-curriculum), and upon curriculum conclusion (post-curriculum).

Section/domain	Survey item	Response options/scale	Administration
Section 1: curricular preferences and baseline needs	Perceived greatest knowledge gap: Which topic do you feel you are furthest away from being at the level of a PGY-2 orthopedic resident?	Practical application of basic sciences; Anatomy and surgical exposure; OR setup and instrumentation; Immobilization proficiency (splints and casts); Consult management; Other (free text)	Pre-curriculum
	Curricular format preference: Do you believe a dedicated, “blocked-off” educational month is superior to a longitudinal curriculum (e.g., one hour per week) for skill acquisition?	Strongly prefer blocked-off month; Slightly prefer blocked-off month; Neutral; Slightly prefer longitudinal or interspersed curriculum; Strongly prefer longitudinal curriculum	Pre-/post-curriculum
	Skill prioritization: If you could only choose one attribute to master by the end of the module, which is most valuable to you?	Manual surgical skills (suturing, reductions); Medical knowledge (anatomy, pathology); Logistical efficiency (consults, OR setup); Patient communication (pre-curriculum) or military-specific topics (post-curriculum); Other (free text)	Pre-/post-curriculum
Section 2: core competency matrix - taking call	1. Independent call management: How confident are you in your ability to work up and manage call patients independently?	Five-point Likert scale*	Pre-, repeat pre-, and post-curriculum
	2. Emergency department hand procedures: How well do you know the steps and overarching goals of emergency department hand procedures (e.g., nail bed repairs, digital blocks, incision and drainage)?	Five-point Likert scale*	Pre-, repeat pre-, and post-curriculum
Section 2: core competency matrix - orthopedic principles	3. Research methodology and administrative requirements: How well do you understand research methodology (e.g., PICOT formatting, manuscript preparation, statistical methods, hospital research resources) and administrative requirements?	Five-point Likert scale*	Pre-, repeat pre-, and post-curriculum
	4. Open surgical procedure proficiency: Overall, how proficient are you in open surgical techniques (surgical approaches, knot tying, suture configurations, soft tissue handling)?	Five-point Likert scale*	Pre-, repeat pre-, and post-curriculum
	5. Arthroscopic surgical proficiency: Overall, how proficient are you in arthroscopic techniques (portal placement, joint entry, diagnostic scope navigation, arthroscopic knots)?	Five-point Likert scale*	Pre-, repeat pre-, and post-curriculum
Section 2: core competency matrix - psychomotor and surgical skills	6. Surgical dexterity and handling: How confident are you in your surgical proprioception, manual dexterity, and instrument handling?	Five-point Likert scale*	Pre-, repeat pre-, and post-curriculum
	7. Surgical anatomy and approaches: How well do you understand surgical anatomy and standard surgical approaches?	Five-point Likert scale*	Pre-, repeat pre-, and post-curriculum
	8. Adult reconstruction: How well do you know the steps and overarching goals of adult reconstruction procedures?	Five-point Likert scale*	Pre-, repeat pre-, and post-curriculum
	9. Elbow procedures: How well do you know the steps and overarching goals of elbow procedures?	Five-point Likert scale*	Pre-, repeat pre-, and post-curriculum
	10. Orthopedic trauma: How well do you know the steps and overarching goals of orthopedic trauma procedures?	Five-point Likert scale*	Pre-, repeat pre-, and post-curriculum
	11. Foot and ankle procedures: How well do you know the steps and overarching goals of foot and ankle procedures?	Five-point Likert scale*	Pre-, repeat pre-, and post-curriculum
	12. Pediatric orthopedics: How well do you know the steps and overarching goals of pediatric orthopedic procedures?	Five-point Likert scale*	Pre-, repeat pre-, and post-curriculum
Section 2: core competency matrix - military surgeon development	13. Deployed military surgeries: How well do you know the operative procedures essential in a deployed military environment (e.g., extremity amputations, fasciotomies, external fixation)?	Five-point Likert scale*	Pre-, repeat pre-, and post-curriculum
	14. Military administrative responsibilities: How well do you understand the military administrative aspects of your role (e.g., military operational requirements, officer duties, military finance)?	Five-point Likert scale*	Pre-, repeat pre-, and post-curriculum
Section 2: core competency matrix - readiness assessment	15. PGY-2 readiness confidence: How confident are you in your ability to function as a competent PGY-2 orthopedic resident today?	Five-point Likert scale*	Pre-, repeat pre-, and post-curriculum
	16. Curricular model efficacy: Rate your overall perception of the educational efficacy of the blocked-off module format compared to longitudinal training.	Five-point Likert scale*	Pre-, repeat pre-, and post-curriculum
Section 3: qualitative feedback	Curricular strengths: What was or will be the most beneficial aspect of this curriculum for junior residents?	Free text	Pre-/post-curriculum, as applicable
	Curricular deficiencies and gaps: What specific orthopedic topics or procedural skills were underrepresented or missing that should be incorporated in future iterations?	Free text	-

**Table 5:** Faculty and senior resident survey instruments † Faculty assessment matrix response scale: 1 = very poor/strongly disagree; 2 = below average/disagree; 3 = competent/neutral; 4 = above average/agree; 5 = exemplary/strongly agree. Administration note: The faculty pre-curriculum survey assessed department priorities and baseline intern expectations. The post-curriculum survey evaluated intern achievement, feasibility impact, and curricular sustainability.

Section/domain	Survey item	Response options/scale	Administration
Section 1: demographics and baseline expectations	Subspecialty role: Which describes your current subspecialty or position?	Trauma; Sports medicine; Adult reconstruction; Hand and upper extremity; Foot and ankle; Spine; Pediatrics; Oncology; General orthopedics; Senior resident (PGY-4, PGY-5, or PGY-6)	Pre-curriculum
	Primary observed intern deficiency: What is the most prominent baseline deficiency observed in incoming interns?	Practical application of basic sciences; Anatomy and surgical exposure; OR setup and instrumentation; Immobilization proficiency (splints and casts); Consult management; Other (free text)	Pre-curriculum
	Format preference: Do you believe a dedicated, “blocked-off” educational month is superior to a longitudinal curriculum for intern skill acquisition?	Strongly prefer blocked-off month; Slightly prefer blocked-off month; Neutral; Slightly prefer longitudinal curriculum; Strongly prefer longitudinal curriculum	Pre-curriculum
	Primary priority attribute: If you could only prioritize mastery of one attribute for an intern prior to PGY-2, which is most valuable?	Manual surgical skills; Medical knowledge; Logistical efficiency; Patient communication; Other (free text)	Pre-curriculum
Section 2: faculty assessment matrix	1. Independent call management: Ability of interns to independently evaluate, triage, and formulate an initial management plan for emergency and inpatient orthopedic consults prior to contacting supervising staff.	Five-point Likert scale†	Pre-/post-curriculum
	2. Research methodology and administration: Understanding of institutional research pathways, IRB protocol development, scientific writing, and research infrastructure.	Five-point Likert scale†	Pre-/post-curriculum
	3. Basic open procedure proficiency: Proficiency in foundational open surgical techniques, including surgical exposures, soft tissue handling, knot tying, and closure.	Five-point Likert scale†	Pre-/post-curriculum
	4. Basic arthroscopic proficiency: Proficiency in foundational arthroscopy, including portal placement, joint entry, diagnostic triangulation, and scope maneuvering.	Five-point Likert scale†	Pre-/post-curriculum
	5. Surgical proprioception and dexterity: Operating room instrument handling, tissue respect, and manual procedural coordination.	Five-point Likert scale†	Pre-/post-curriculum
	6. Anatomy and surgical approaches: Comprehension of anatomic intervals, standard surgical exposures, and preservation of neurovascular structures at risk.	Five-point Likert scale†	Pre-/post-curriculum
	7. Overall military responsibilities: Grasp of military officer duties, administrative workflows, operational finance, and deployed surgical readiness (e.g., fasciotomies, external fixation, emergent amputations).	Five-point Likert scale†	Pre-/post-curriculum
	8. PGY-2 readiness confidence: Overall confidence in the intern cohort's readiness to assume junior resident responsibilities and independent on-call duties.	Five-point Likert scale†	Pre-/post-curriculum
Section 3: feasibility, logistics, and qualitative assessment	Teaching participation volume: How many instructional sessions did you teach?	0; 1; 2; 3 or more	Post-curriculum
	Clinical scheduling disruption: Did you have to reschedule or cancel clinical duties (clinic/OR) to participate in teaching?	Yes, canceled or rescheduled clinical work; No, utilized existing non-clinical time; Not applicable, did not teach	Post-curriculum
	Curricular worth and sustainability: If clinical adjustments were made, was the educational impact worth the scheduling disruption, and would you participate again?	Yes, worth it and would participate again; No, not worth it; Conditional/depends	Post-curriculum
	Endorsement of military content: The enhanced focus on military-specific operational and administrative skills was valuable and should be maintained in future years.	1 = strongly disagree to 5 = strongly agree	Post-curriculum
	Curricular strengths: What was the most beneficial aspect of this curriculum for the intern cohort?	Free text	Post-curriculum
	Curricular deficiencies and gaps: What specific orthopedic topics or technical skills remain underrepresented and should be added or expanded next year?	Free text	-
